# Influence of Soil Type and Moisture on Pupal Development of *Chrysomya rufifacies* (Macquart) at Two Different Temperatures

**DOI:** 10.3390/insects15070479

**Published:** 2024-06-27

**Authors:** Tharindu B. Bambaradeniya, Paola A. Magni, Ian R. Dadour

**Affiliations:** 1 School of Medical, Molecular & Forensic Sciences, Murdoch University, Murdoch, WA 6150, Australia; sritharindu27@gmail.com (T.B.B.); p.magni@murdoch.edu.au (P.A.M.); 2 Harry Butler Institute, Murdoch University, Murdoch, WA 6150, Australia; 3 Source Certain, P.O. Box 1570, Wangara, WA 6947, Australia

**Keywords:** myiasis, forensic entomology, pupariation, pupation

## Abstract

**Simple Summary:**

*Chrysomya rufifacies* (Macquart), commonly known as the hairy maggot blow fly, is important in forensic and medical contexts because it colonizes carcasses and is a causative agent of myiasis in animals and humans. Studying the development of its pupae is crucial for understanding the time since death in forensic investigations and for containing the spread of myiasis. This study investigated how temperature, soil type, and moisture affect the development of *Ch. rufifacies* pupae in Western Australia.

**Abstract:**

The present study investigates the developmental process of *Chrysomya rufifacies* (Macquart) pupae and their dependency on soil composition, moisture levels, and temperature changes. This research holds implications for forensic and veterinary applications, providing crucial insights for estimating minimum postmortem intervals and managing myiasis-causing flies in diverse environments. Specifically, the study explores the impact of five moisture content levels in loam and sandy soils (0%, 20%, 40%, 60%, and 80%) on the pupal development of *Ch. rufifacies* under two distinct constant temperature regimes (24 ± 1 °C and 30 ± 1 °C). A significant correlation was observed between soil type and temperature regarding the time required to complete the pupal stages; however, moisture had no significant impact. Larvae exhibited varying survival rates across the two temperatures and five moisture levels in the two types of soils, particularly under extremely lower moisture conditions (0%) at 30 ± 1 °C, failing to progress to the pupariation stage. Additionally, growth parameters such as pupal length and width of the fully formed puparia were significantly impacted by temperature, soil type, and moisture level. Adult head width was systematically measured across different moisture levels and soil types, revealing distinct temperature-dependent responses. Furthermore, a sex-specific analysis highlighted that female *Ch. rufifacies* consistently displayed larger head widths and higher emergence rates compared to their male counterparts. This research enhances our understanding of the intricate interrelationship among three environmental variables: soil type, moisture level, and temperature, elucidating their collective impact on the pupation processes of dipterans.

## 1. Introduction

*Chrysomya rufifacies* (Macquart) (Diptera: Calliphoridae) is widely recognized for its veterinary and forensic importance, as it is involved in carrion decomposition and causing myiasis in animals [1] and humans [2]. Typically, *Ch. rufifacies* is considered a secondary species that arrives at carcasses and infests wounds that have already been colonized by primary species [2,3]. Being a holometabolous insect [4], the larvae develop through three instars, and during the final instar, they cease feeding and they begin to wander in search of a suitable location to pupate [5]. In an outdoor setting, the most likely medium they encounter is soil [6].

Following this wandering period, the pupariation (i.e., formation of the puparium from the last larval skin, which hardens and darkens to protect the pupa inside), pupation (i.e., the process of metamorphosis by which a larva transforms into a pupa), and adult emergence success rates of dipterans depend on the type and structure of the soil in which they burrow prior to pupation [4,5]. The soil type and structure are intricately linked to drainage, moisture content, hygroscopicity, air retention capacity, vegetation, and temperature. Soil moisture is a crucial factor impacting the pupation behavior of hypodermal blow fly pupae, with two extremes—dry and wet soil—being detrimental to their development [6].

Several studies have explored the influence of soil moisture on the pupation behavior of dipterans. The earliest investigation was conducted by Melvin and Bushland (1938) [7], who examined the impact of alkalinity, acidity, and moisture content on the adult emergence percentages of *Cochliomyia americana* (Cush. and Patt.). They placed puparia in sand saturated at different percentages of distilled water or with various dilutions of sulfuric acid or potassium hydroxide. Bruce (1939) [4] expanded this research by examining the relationship between soil moisture and adult emergence for *Haematobia irritans* (L.), *Musca domestica* (L.), and *Co. americana* in fine sandy soil with moistures ranging between 0 and 16%. Another study, conducted by Kokdener and Yurtgan (2022) [6], is the only available laboratory-based comparative study on the influence of soil moisture content (0%, 25%, 50%, 75%, and 100%) on the pupal development of *Lucilia sericata* (Meigen) in three types of soils: clay, loamy, and sandy. Pitts and Wall (2005) [8] contributed to this field by studying the same species in the United Kingdom, recording pupariation and adult emergence success rates at two soil moisture content levels: 26.3% and 21.2% under field conditions. Two additional studies investigated the impact of different temperature conditions on pupal development, which focused on soil type and temperature effect on pupation; however, the moisture content of the soil was not considered in these studies. Dallwitz (1984) [9] explored the pupation rate and survival of pupae of *L. cuprina* (Wiedemann) under varying fluctuating (9.5 °C to 30.4 °C) and constant temperatures (15 °C, 20 °C, 25 °C, 28 °C, 30 °C, 31 °C, 33 °C, and 35 °C) in air-dried soil, while Kotze et al. (2015) [10] recorded similar pupal development parameters at temperatures ranging from 18 °C to 33 °C in dry sand. The current study addresses all three factors, including temperature, soil type, and moisture content.

The primary objective of the present study was to explore the pupariation and pupation behavior of *Ch. rufifacies* within two distinct soil types prevalent in Western Australia: loam and sand. This investigation encompassed five different soil moisture conditions, conducted at two consistent temperatures, namely 24 ± 1 °C and 30 ± 1 °C, representing autumn and summer temperatures in Western Australia. The study focused on various aspects of the pupariation and pupation behavior of *Ch. rufifacies.* These include the development rate of pupation, success rate of pupariation and pupation, morphological changes of pupae, length and width variations of the fully formed puparia, and changes in the head width of emerging male and female adults. This study provides valuable insights that may be helpful for the forensic entomologist in determining minimum postmortem intervals and the veterinary practitioner in managing myiasis.

## 2. Materials and Methods

### 2.1. Source Colony Maintenance and Production of Post Feeding 3rd Instar Larvae

The *Ch. rufifacies* colonies were generated and maintained in an insectary, following the protocols outlined in Bambaradeniya et al. (2023) [11]. Egg batches, acquired from the oviposition medium within these colonies, were relocated simultaneously onto 200 g of swine skeletal muscle from the thigh flesh of a domestic pig (*Sus scrofa domesticus* L.) (purchased from a local butcher shop from one pig) using a fine-tipped artist paintbrush. Subsequently, these muscle samples and egg batches were positioned in ventilated glass containers on top of a 3 cm layer of sand. The containers housing the egg batches were then relocated to a growth chamber (Fisher & Paykel^®^ with a purpose-built temperature and humidity regulator) after being covered with a fine mesh cover. The growth chamber conditions were consistently maintained at temperatures of 24 ± 1 °C and 30 ± 1 °C, respectively. The relative humidity (RH) and photoperiod were set at 70% and 12:12 L:D for both temperatures, respectively. The larvae underwent development within the glass containers, progressing until reaching the post-feeding 3rd instar stage before being removed for pupal rearing on soils of varying moisture levels.

### 2.2. Setting Up Containers with Two Soil Types at Five Moisture Levels

The study involved using approximately 2 kg of each soil type (loam and sandy) for both temperatures. The loam soil was collected near the sheep farm at Murdoch University, Perth, and the sandy soil used was construction-grade soil. Each soil type was sieved to remove debris, spread out, and sun-dried for seven days prior to the experiment. Twenty plastic containers (400 mL) were filled with 100 g (VEVOR Lab Analytical Balance^®^) of loam or sandy soil for each of the four replicate studies (n = 4). The number of replicates for each moisture and soil type combination at a single temperature regime was determined based on guidelines from previous publications [6]. The initial moisture content of each soil sample was measured with a moisture meter (Yieryi PMS710^®^- Shanghai, China). Distilled water was added carefully to each container to achieve five predetermined moisture levels: 0%, 20%, 40%, 60%, and 80%, using the moisture meter. The final weight of each container was recorded. The accuracy of the moisture levels was confirmed by a predetermined equation: moisture content (%) = [weight of distilled water/(weight of saturated soil − weight of dried soil)] × 100% [12]. Soil was used once and then discarded.

### 2.3. Transferring Post-Feeding Third Instar Larvae to Each Container

**Figure 1:**
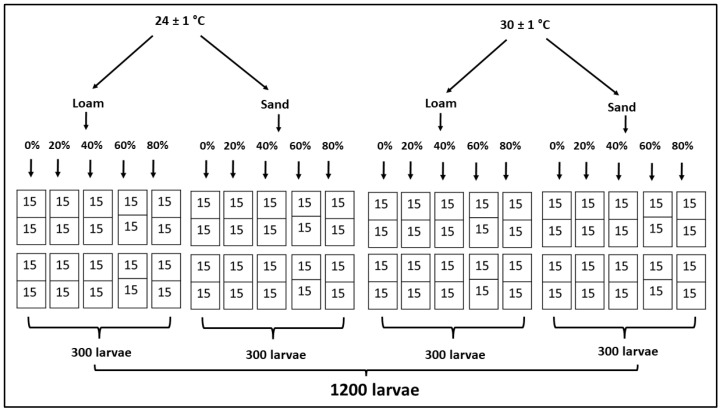
*Ch. rufifacies* larval number arrangement at two temperatures: 24 ± 1 °C or 30 ± 1 °C: two soil types: loam and sand; and five moisture levels: 0%, 20%, 40%, 60%, and 80%.

Larvae reared under each temperature regime were carefully extracted from their respective rearing containers, and their developmental stage (post feeding 3rd instar stage) was determined by assessing their body size and wandering behavior [11]. A total of 15 larvae were collected and applied to a single replicate of each soil type and temperature. Therefore, to accommodate two soil types at two temperatures over five soil moisture conditions (each moisture condition replicated twice), the total number of larvae utilized for the entire experiment was approximately 1200 (4 (replicates) × (2 (soil types) × 15 (number of larvae) × 5 (moisture levels) × 2 (temperatures)) (Figure 1).

In each container, larvae were transferred onto a specific soil type (sand or loam), and the container (400 mL) was covered with a cloth mesh and firmly secured with a rubber band. In total, 40 replicates were set up for two soil types at each temperature, resulting in a total container count of 80 across both temperatures (Figure 1). All the containers were placed inside a growth chamber, and the temperature was set at either 24 ± 1 °C or 30 ± 1 °C. The relative humidity (RH) and photoperiod inside the growth chamber were set at 70% and 12:12h L:D for both temperature regimes.

### 2.4. Data Gathering

Observations were conducted every three hours on the containers placed in the incubator to document the initiation of pupariation. Following the onset of pupariation, one puparium was extracted at eight-hour intervals from each container. Subsequently, these puparia were punctured and then subjected to a hot water kill before being preserved in 70% alcohol [13]. The length and width of preserved puparia were measured using vernier calipers (Baker DC20^®^). They were then dissected using a scalpel, and the developmental landmarks of the pupae were documented after capturing images with a digital camera. The complete duration of pupation for the two temperature regimes was categorized into four distinctive landmark stages: the pre-cryptocephalic pupal stage, the cryptocephalic pupal stage, the phanerocephalic pupal stage, and the pharate adult stage. The pre-cryptocephalic pupal stage was characterized by the larval–pupal apolysis, where the pre-pupa remained tightly affixed to the inner surface of the puparium while retaining most of the larval features, including body segmentation and the presence of the cephalopharyngeal skeleton. Subsequently, the cryptocephalic pupal stage ensued, marked by the formation of legs and wings. Following this, the phanerocephalic pupal stage emerged, distinguished by the clear separation of the pupal body into three segments: head, thorax, and abdomen. Ultimately, the pharate adult stage represented the form of an adult enclosed within the puparium, where the epidermal cells were distinct from the pupal cuticle. This classification aligns with established criteria outlined by Greenberg and Kunich (2002) [14], Li et al. (2023) [15], and Bambaradeniya et al. (2024) [16].

At the 48h mark from the commencement of pupariation, the total number of puparia in each container was recorded. The time of adult emergence was recorded through continuous observation. The number of emerged adults was recorded 48 h after the first emergence in each container.

Five emerged adults from each container were sacrificed using a hot water kill [13], transferred to separate containers, and stored in a refrigerator for a minimum of two days when measurements were obtained. The sex of the flies was determined by their interocular distance, and their size was calculated using head widths measured using vernier calipers.

### 2.5. Data Analysis

The data analysis was conducted using R Studio version 4.3.3 software. Initially, the assumption of normality was assessed using the Shapiro–Wilk test. Given the non-normal distribution of certain variables, non-parametric tests were employed for calculations related to puparia numbers and adult numbers. Parametric tests were used for analysing the remaining variables. A significance level of 0.05 was applied to all analyses. The interaction between temperature, soil type, and moisture in the pupation period was analyzed using a three-way ANOVA. The assessment of puparia numbers and adult numbers across different soil types, temperatures, and moisture levels was performed using the aligned rank transformation ANOVA followed by a post hoc comparison: aligned rank transformation contrast. Pupal length and width changes were analyzed using a three-way MANOVA (Multivariate Analysis of Variance). To elucidate the response of female and male adult head width changes to the combined effects of temperature, soil type, and moisture, generalized additive models (GAMs) were utilized. Graphical representations of the data were generated using the same statistical software.

## 3. Results

### 3.1. Pupation Periods

**Figure 2:**
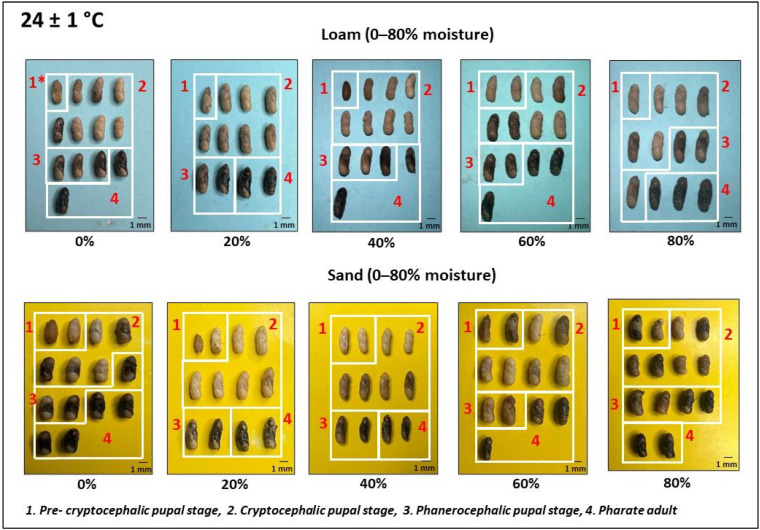
Morphological changes (lateral view) of pupation landmarks of *Ch. rufifacies* in loam and sand at 24 ± 1 °C.

**Figure 3:**
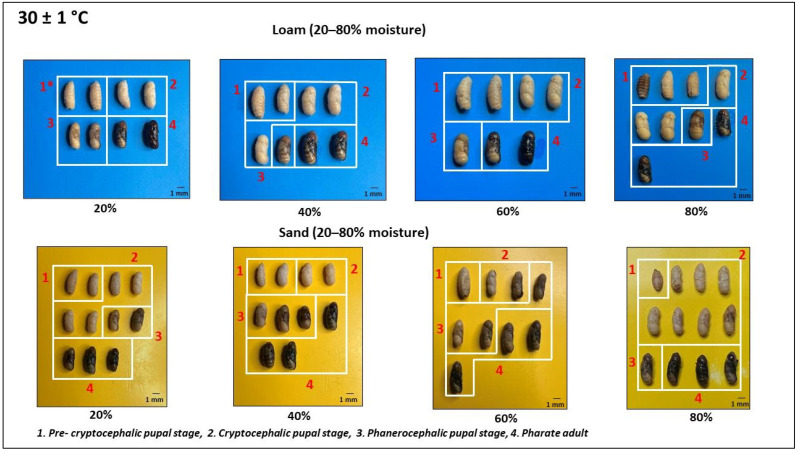
Morphological changes (lateral view) of pupation landmarks of *Ch. rufifacies* in loam and sand at 30 ± 1 °C.

**Figure 4:**
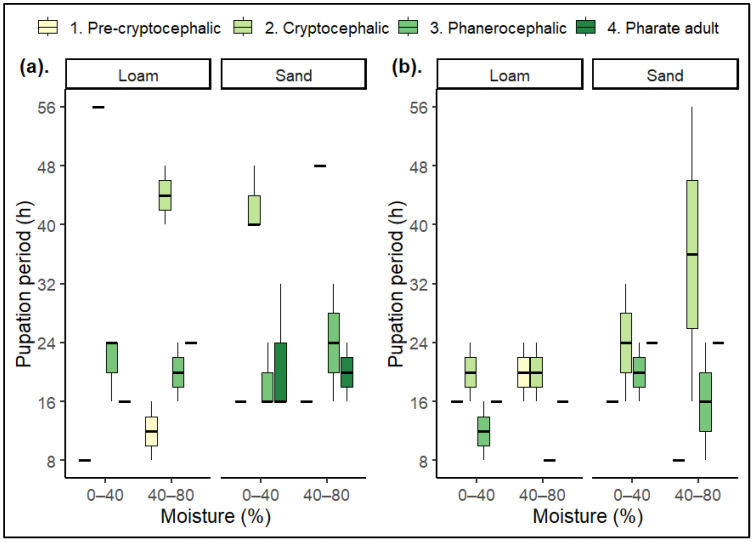
Pupation periods (period ± SD) of pupation landmarks of *Ch. rufifacies* in loam and sand at 24 ± 1 °C (**a**) and 30 ± 1 °C (**b**) in two moisture ranges: 0–40% and 40–80%.

Observations based on pupation landmark stages of preserved *Ch. rufifacies* puparia at 24 ± 1 °C and 30 ± 1 °C were used to create separate diagrams illustrating the pupation process in each soil type at different moisture levels (Figure 2 and Figure 3). Based on these diagrams, the time taken to achieve each landmark stage was determined and summarized under two moisture ranges: 0–40% and 40–80% (Table 1, Figure 4).

The overall pupation rate varied across all three variables: temperature, soil type, and moisture ranges, showing faster and slower rates with no specific relationship with moisture differences. However, pupation time did vary with temperature and soil type (Table 1). The statistical analysis revealed that soil type (df = 1, 60; F = 7.06, *p* = 0.02) and temperature (df = 1, 60; F = 49.42, *p* ≤ 0.001) demonstrated a significant influence on the pupation period. However, moisture levels did not significantly impact the pupation period (df = 1, 60; F = 0.13, *p* = 0.73). None of the interactions between temperature and soil type (df = 1, 60; F = 4.32, *p* = 0.06), temperature and moisture level (df = 1, 60; F = 0.12, *p* = 0.75), and moisture level and soil type (df = 1, 60; F = 0.28, *p* = 0.60) were found to be statistically significant.

Overall, the combined effect of temperature, soil type, and moisture level did not significantly influence the pupation period (df = 1, 60; F = 0.24, *p* = 0.63).

### 3.2. Rates of Pupation and Adult Emergence

The highest mean pupation occurred in loam soil at 24 ± 1 °C at zero moisture content (14.5 ± 0.57). The greatest mean adult emergence number was observed in loam (7 ± 1.82) and sand (7 ± 1.82) at 24 ± 1 °C with 80% moisture content (Table 2). The soil type (df = 1, 60; F = 4.46, *p* = 0.038), moisture (df = 4, 60; F = 73.79, *p* ≤ 0.001), and temperature (df = 1, 60; F = 25.93, *p* ≤ 0.001) alone exert a significant impact on pupation. In addition, the interaction between moisture and soil type (df = 4, 60; F = 45.47, *p* ≤ 0.001), moisture and temperature (df= 4, F = 67.72, *p* ≤ 0.001), and temperature and soil type (df = 1, 60; F = 95.56, *p* ≤ 0.001) demonstrated a significant influence on pupation. Overall, all three independent variables (temperature, soil type, and moisture) have an impact on pupation (df = 4, 60; F = 21.01, *p* ≤ 0.001).

A significant impact on adult emergence was detected between the interaction of temperature and moisture (df = 4, 60; F = 19.34, *p* ≤ 0.001) but not between moisture and soil type (df = 4, 60; F = 0.65, *p* = 0.63) and temperature and soil type (df = 1, 60; F = 0.56, *p* = 0.46). Individually, variations of temperature (df = 1, 60; F = 24.74, *p* ≤ 0.001) and moisture (df = 4, 60; F = 38.77, *p* ≤ 0.001) were found to impact adult emergence, but not soil type (df = 1, 60; F = 0.69, *p* = 0.41). Overall, the collective interaction between temperature, soil type, and moisture level were not found to be significant (df = 4, 60; F = 0.75, *p* = 0.56).

### 3.3. Length and Width of the Puparia

The maximum length recorded for all preserved puparia were those reared in loam at 30 ± 1 °C (10.27 mm), and the minimum was in sand at the same temperature (6.75 mm). A similar trend was observed in the width of the puparia, with maximum and minimum values recorded in loam (4.33 mm) and sand (2.57 mm) at 30 ± 1 °C, respectively (Table 3 and Table 4).

The MANOVA results indicated that only temperature (df = 1, 60; F = 1689.36, *p* ≤ 0.001) and moisture (df = 4, 60; F = 60.95, *p* ≤ 0.001) had a significant impact on the length and width change, while soil type (df = 1, 60; F = 2.50, *p* = 0.085) had no significant effect. When considering combinations, the interaction between temperature and moisture (df = 4, 60; F = 58.26, *p* ≤ 0.001), soil and moisture (df = 4, 60; F = 4.71, *p* ≤ 0.001), and temperature and soil (df = 1, 60; F = 3.65, *p* = 0.028) demonstrated an impact on the length and width change in puparia. In summary, all three independent variables, temperature, soil type, and moisture, collectively contributed to the observed changes in the length and width of the puparia (df = 4, 60; F = 3.48, *p* ≤ 0.001).

### 3.4. Growth Parameters of Male and Female Adult Flies Head Width

The female percentages (preserved to measure head width) in loam and sand at 24 ± 1 °C were 43.4% and 42.5%, respectively, while at 30 ± 1 °C, they were 40% and 33.75% (based on 200 adult flies at each temperature regime). The maximum head width (3.28 mm) within any sex was recorded in sand at 24 ± 1 °C, and the minimum (2.2 mm) was in loam at 30 ± 1 °C. Furthermore, within moisture levels, the highest mean female head width was recorded in sandy soil at 24 ± 1 °C with 60% moisture (3.70 ± 0.09 mm), whereas for males, this was recorded in loam at 24 ± 1 °C with 20% moisture (3.52 ± 0.10 mm) (Table 5).

GAM models revealed that the head width of adults varied despite the sex based on soil type change (Table 6).

## 4. Discussion

In the present study, the pupation rate of *Ch. rufifacies* was examined at two different temperatures for loam and sand across 0–40% and 40–80% moisture ranges. Regarding the pupation period, the study found that the pupation rate was faster at 30 ± 1 °C compared to 24 ± 1 °C. At both temperatures, loam facilitated faster pupation than sand. However, a specific relationship between moisture levels and the acceleration or deceleration of the pupation period was not detected. This observation is consistent with the study by Kökdener and Yurtcan (2022) [6], where pupation in *L. sericata* was faster in loam than in sand at 27 °C, but no acceleration or deceleration effect on the pupation period was found due to moisture levels. The present study concluded that moisture did not significantly impact the pupation period of *Ch. rufifacies*. However, the two different temperatures, 24 ± 1 °C and 30 ± 1 °C, showed an impact, with soil types affecting the pupation period. According to a recent study by Kökdener and Yurtcan (2022), at 27 °C, the moisture levels in sandy, clay, and loamy soils have a significant impact on the pupation period of *L. sericata*.

The pupariation rate and the number of pupae that eclosed within 48 hrs from their first appearance were recorded. Consistent with the findings of Kökdener and Yurtcan (2022) [6], higher pupariation and pupation rates were observed at lower moisture levels for both *Ch. rufifacies* and *L. sericata* in loam. However, *Ch. rufifacies* failed to pupate at 0% moisture in both sand and loam. It was also observed that larvae exposed to this moisture level and temperature died due to dehydration. In contrast, *L. sericata* exhibited high pupal survival at 0% moisture in the sand. This suggests species-specific responses to moisture levels, as well as keeping in mind that the disparities between these two studies can be attributed to variations in experimental parameters, such as temperature (27 °C in Kökdener and Yurtgan’s study [6] and 24 ± 1 °C and 30 ± 1 °C in the current study) and relative humidity (65% in Kökdener and Yurtgan’s study [6] and 70% in the present study) within the growth chamber.

The current investigation also documents the external morphological changes of *Ch. rufifacies* pupae. This study holds significant importance, as it is the first to record and analyze these morphological alterations in response to three key environmental factors: soil type, soil moisture, and temperature.

In the current study, the growth parameters examined for puparia and adults in relation to soil type and moisture change included the length and width of puparia, as well as the head width of adults. Kökdener and Yurtgan (2022) [6] recorded pupal and adult weight for *L. sericata*, while Bauer et al. (2020) [17] recorded only the body weight of *Co. macellaria* larvae. Previous studies have recognized head width as a meaningful growth parameter for dipterans, as it has been associated with various factors such as substrate location [18], larval density [19], and temperature change [20]. The present study revealed that at 24 ± 1 °C, both male and female head widths were slightly wider in conditions of moderate moisture (40–80%) in both loam and sand. However, at 30 ± 1 °C, no distinct changes in head width were observed across all moisture levels and soil types. These findings align with the study by Ong et al. (2018) [21] at 25 °C, where head widths of *Megaselia scalaris* (Loew) were influenced by substrate moisture levels greater than 60%. In the current study, the results showed that, on average, females had a wider head width compared to males in 13 of the 20 replicates. This finding was also supported by Kökdener’s (2021) [22] study, which found that female *M. domestica* had wider head widths than males when reared on three different food types (wheat bran, fish meal, and poultry meal) at varying moisture levels (50%, 60%, 70%, 80%, and 90%).

The recorded female-to-male ratios in this study at 24 ± 1 °C were 43.4:56.6 in loam and 42.5:57.5 in sand. At 30 ± 1 °C, the ratios were 40:60 for loam and 33.8:66.2 for sand. In all cases, female emergence exceeded male emergence. In contrast, Kökdener and Yurtgan (2022) [6] reported higher female-to-male ratios for *L. sericata* in sand at 54.4:45.6 and in loam at 52.4:47.6. On the other hand, Bauer et al. (2020) [17] presented results contrary to the present study, reporting lower female-to-male ratios of 46.5:53.5 and 27.1:72.9 when *Co. macellaria* was fed with beef liver at moisture levels of 50% and 70.8%. However, it is important to note that these moisture levels pertained to the feeding medium, not the pupation medium.

The results highlight the crucial significance of soil type and moisture levels in influencing pupation development and subsequent adult emergence. This factor becomes critically important within the forensic context when diverse soil conditions and varying moisture levels can exert a substantial impact on the developmental trajectories of larvae. This may potentially compromise the precision of estimated minimum postmortem intervals. Furthermore, this correlation between soil moisture content may have some impact on the incidence of myiasis infestations. Field observational data indicates that, during periods of significant rainfall, myiasis infestations were more prevalent in livestock situated on well-drained soils, contrasting the relative scarcity of myiasis in livestock grazing on wet soils [5]. Conversely, during the dry season, a discernible increase in screwworm cases was observed in regions characterized by poorly drained soils. This suggests that dry soils may contribute to myiasis control by subjecting the larvae to elevated ambient temperatures, particularly in regions with high humidity [5,6].

## Figures and Tables

**Table 1:** Pupation periods (h) of *Ch. rufifacies* larvae in loam and sand at 24 ± 1 °C and 30 ± 1 °C across two moisture ranges: 0–40% and 40–80% (n = 4, mean ± SD, standalone numbers had no SD). (P—pre-cryptocephalic, C—cryptocephalic, Ph—phanerocephalic, PA—pharate adult).

Temperature (°C)	Soil Type	Moisture Range (%)	Pupation Time (h)	Pupation Landmark (h)			
				P	C	Ph	PA
24 ± 1 °C	loam	0–40	101.30 ± 4.61	8.00	56.00	21.33 ± 4.61	16.00
		40–80	100.00 ± 5.65	12.00 ± 5.65	44.00 ± 5.65	20.00 ± 5.65	24.00
	sand	0–40	101.30 ± 9.23	16.00	42.60 ± 4.61	18.60 ± 4.61	21.33 ± 9.23
		40–80	108.00 ± 5.65	16.00	48.00	24.00 ± 11.31	20.00 ± 5.65
30 ± 1 °C	loam	0–40	64	16.00	20.00 ± 5.65	12.00 ± 5.65	16.00
		40–80	64.00 ± 11.31	20.00 ± 5.65	20.00 ± 5.65	8.00	16.00
	sand	0–40	84.00 ± 5.65	16.00	24.00 ± 11.31	20.00 ± 5.65	24.00
		40–80	84.00 ± 16.97	8.00	36.00 ± 28.28	16.00 ± 11.31	24.00

**Table 2:** Mean number of puparia and emerged adults (Mean no. ± SD, n = 4) of *Ch. rufifacies* after 48h since the first record of pupariation and emergence in sandy and loam soil at 24 ± 1 °C and 30 ± 1 °C across five moisture contents; 0, 20%, 40%, 60%, and 80%. * Means are significantly different by aligned rank transformation contrasted at the 5% level of significance.

Temperature (°C)	Soil Type	Moisture Content (%)	Mean No. of Puparia	Mean No. of Adults
24 ± 1 °C	loam	0	14.50 ± 0.57 *	2.50 ± 1.29 *
		20	12.00 ± 2.16 *	5.25 ± 0.50*
		40	5.25 ± 1.50	5.50 ± 0.57 *
		60	13.25 ± 2.21	4.75 ± 0.50 *
		80	6.00 ± 0.81 *	7.00 ± 1.82 *
	sand	0	6.50 ± 0.57 *	4.00 ± 1.15 *
		20	6.25 ± 0.50	5.25 ± 0.50 *
		40	10.75 ± 0.50 *	5.50 ± 0.57 *
		60	10.25 ± 1.89	4.75 ± 0.50 *
		80	3.75 ± 0.95 *	7.00 ± 1.82 *
30 ± 1 °C	loam	0	0 *	0 *
		20	12.25 ± 0.95 *	4.25 ± 0.50 *
		40	7.75 ± 0.95 *	6.0 ± 0 *
		60	8.50 ± 0.57 *	5.5 ± 0.57
		80	4.75 ± 0.50	5.75 ± 0.95
	sand	0	0 *	0 *
		20	8.50 ± 0.57 *	4.25 ± 0.50 *
		40	10.75 ± 0.95 *	6.0 ± 0 *
		60	11.75 ± 2.21 *	5.5 ± 0.57 *
		80	13.00 ± 0.81*	6.0 ± 0.81 *

**Table 3:** Maximum, minimum, and mean length (Mean ± SD, n = 4) of *Ch. rufifacies* puparia reared in sandy and loam soil at 24 ± 1 °C and 30 ± 1 °C across five moisture contents: 0, 20%, 40%, 60%, and 80%.

Temperature (°C)	Soil Type	Maximum Length (mm)	Minimum Length (mm)	Mean Length (mm ± SD)	Moisture Level	Mean Length (mm ± SD)
24 ± 1 °C	Loam	9.72	7.44	8.61 ± 0.54	0	8.47 ± 0.43
					20	8.63 ± 0.39
					40	8.56 ± 0.35
					60	9.32 ± 0.31
					80	8.04 ± 0.28
	Sand	9.72	7.19	8.59 ± 0.55	0	8.64 ± 0.47
					20	8.89 ± 0.35
					40	8.26 ± 0.38
					60	9.09 ± 0.40
					80	8.05 ± 0.39
30 ± 1 °C	Loam	10.27	7.39	8.35 ± 0.78	0	-
					20	7.9 ± 0.34
					40	8.1 ± 0.29
					60	8.06 ± 0.28
					80	9.42 ± 0.89
	Sand	9.37	6.75	7.88 ± 0.54	0	-
					20	7.49 ± 0.58
					40	8.01 ± 0.36
					60	7.97 ± 0.35

**Table 4:** Maximum, minimum, and mean width (mean ± SD, n = 4) of *Ch. rufifacies* puparia reared in sandy and loam soil at 24 ± 1 °C and 30 ± 1 °C across five moisture contents: 0, 20%, 40%, 60%, and 80%.

Temperature (°C)	Soil Type	Maximum Width (mm)	Minimum Width (mm)	Mean Width (mm ± SD)	Moisture Level	Mean Width (mm ± SD)
24 ± 1 °C	Loam	4.02	2.9	3.57 ± 0.221	0	3.67 ± 0.15
					20	3.69 ± 0.20
					40	3.54 ± 0.18
					60	3.58 ± 0.19
					80	3.35 ± 0.22
	Sand	4.09	2.46	3.55 ± 0.298	0	3.63 ± 0.13
					20	3.78 ± 0.15
					40	3.46 ± 0.13
					60	3.70 ± 0.37
					80	3.18 ± 0.19
30 ± 1 °C	Loam	4.33	2.8	3.31 ± 0.393	0	-
					20	3.11 ± 0.18
					40	3.22 ± 0.15
					60	3.09 ± 0.17
					80	3.86 ± 0.41
	Sand	3.77	2.57	3.20 ± 0.254	0	-
					20	3.06 ± 0.27
					40	3.18 ± 0.18
					60	3.20 ± 0.27
					80	3.37 ± 0.19

**Table 5:** Maximum, minimum, and mean head with (mean ± SD, n = 4) of *Ch. rufifacies* adult reared in sandy and loam soil at 24 ± 1 °C and 30 ± 1 °C across five moisture contents: 0, 20%, 40%, 60%, and 80%.

Temperature (°C)	Soil Type	Max Width (mm)	Minimum Width (mm)	Mean Width (mm ± SD)	Moisture (%)	Male (Mean ± SD)	Female (Mean ± SD)
24 ± 1 °C	loam	3.7	2.36	3.29 ± 0.22	0	3.10 ± 0.19	3.42 ± 0.14
					20	3.52 ± 0.08	3.39 ± 0.14
					40	3.28 ± 0.14	3.53 ± 0.07
					60	3.49 ± 0.01	3.41 ± 0.13
					80	3.02 ± 0.19	3.14 ± 0.22
	sand	3.83	2.70	3.32 ± 0.23	0	3.19 ± 0.15	3.15 ± 0.13
					20	3.52 ± 0.11	3.48 ± 0.20
					40	3.24 ± 0.11	3.28 ± 0.14
					60	3.48 ± 0.19	3.70 ± 0.10
					80	3.08 ± 0.15	3.21 ± 0.14
30 ± 1 °C	loam	3.45	2.20	2.71 ± 0.19	0	-	-
					20	2.58 ± 0.19	2.72 ± 0.18
					40	2.74 ± 0.16	2.71 ± 0.26
					60	2.68 ± 0.12	2.72 ± 0.08
					80	2.71 ± 0.14	2.88 ± 0.26
	sand	3.2	2.29	2.68 ± 0.19	0	-	-
					20	2.58 ± 0.15	2.81 ± 0.15
					40	2.76 ± 0.19	2.80 ± 0.23
					60	2.62 ± 0.18	2.75 ± 0.17
					80	2.57 ± 0.10	2.83 ± 0.27

**Table 6:** Estimated regression parameter, smoother, t-value, and *p*-values of the final model describing head width change of *Ch. rufifacies* in loam and sand at 24 ± 1 °C and 30 ± 1 °C.

Soil Type and Temperature	Factor	Estimate	SE	t-Value	Pr (>|t|)
Loam (24 ± 1 °C)	Moisture	−0.002	0.001	−2.45	0.02
	Male	−0.173	0.040	−4.26	4.72 × 10^−5^
	Female	-	-	-	-
Sand (24 ± 1 °C)	Moisture	−4.287 × 10^−5^	8.346 × 10^−4^	−0.05	0.96
	Male	−6.206 × 10^−2^	4.768 × 10^−2^	−1.30	0.20
	Female	-	-	-	-
Loam (30 ± 1 °C)	Moisture	0.002	0.001	2.26	0.03
	Male	−0.094	0.041	−2.26	0.03
	Female	-	-	-	-
Sand (30 ± 1 °C)	Moisture	−0.001	0.001	−0.78	0.44
	Male	−0.164	0.0424	−3.87	0.001
	Female	-	-	-	-

## Data Availability

The data that supports the findings of this study are available from the corresponding author upon reasonable request.

## References

[1] Bambaradeniya Y.T.B., Karunaratne W.A.I.P., Tomberlin J.K., Magni P.A. (2021). Effect of type of tissue on the development of *Chrysomya rufifacies* (Diptera: Calliphoridae) in Sri Lanka. J. Med. Entomol..

[2] Hall M.J., Smith K.G. (1993). Diptera causing myiasis in man. Medical Insects and Arachnids.

[3] Lang M.D., Allen G.R., Horton B.J. (2006). Blowfly succession from possum (*Trichosurus vulpecula*) carrion in a sheep-farming zone. Med. Vet. Entomol..

[4] Bruce W.G. (1939). Some observations on insect edaphology. J. Kans. Entomol. Soc..

[5] Norris K.R. (1965). The bionomics of blow flies. Annu. Rev. Entomol..

[6] Kökdener M., Yurtgan Ş.M. (2022). The effect of soil type and moisture level on the development of *Lucilia sericata* (Diptera: Calliphoridae). J. Med. Entomol..

[7] Melvin R., Bushland R.C. (1938). Effects of acidity, alkalinity and moisture content of the soil on emergence of *Cochliomyia americana* C. & P. J. Econ. Entomol..

[8] Pitts K.M., Wall R. (2005). Winter survival of larvae and pupae of the blowfly, *Lucilia sericata* (Diptera: Calliphoridae). Bull. Entomol. Res..

[9] Dallwitz R. (1984). The influence of constant and fluctuating temperatures on development rate and survival of pupae of the Australian sheep blowfly *Lucilia cuprina*. Entomol. Exp. Appl..

[10] Kotzé Z., Villet M.H., Weldon C.W. (2015). Effect of temperature on development of the blowfly, *Lucilia cuprina* (Wiedemann). Int. J. Legal Med..

[11] Bambaradeniya Y.T.B., Magni P.A., Dadour I.R. (2023). Current status of five warm season Diptera species in estimating the post-mortem interval. Ann. Entomol. Soc. Am..

[12] Chen M., Shelton A.M. (2014). Impact of soil type, moisture, and depth on swede midge (Diptera: Cecidomyiidae) pupation and emergence. Environ. Entomol..

[13] Bambaradeniya T.B., Magni P.A., Dadour I.R. (2023). A Summary of Concepts, Procedures and Techniques Used by Forensic Entomologists and Proxies. Insects.

[14] Greenberg B., Kunich J.C. (2002). Entomology and the Law: Flies as Forensic Indicators.

[15] Li L., Zhang Y., Chen Y., Guo Y., Wang Y., Hu G., Kang C., Wang J., Wang Y. (2023). Intrapuparial development and age estimation of *Sarcophaga peregrina* (Diptera: Sarcophagidae) for postmortem interval estimation. J. Asia-Pac. Entomol..

[16] Bambaradeniya T.B., Magni P.A., Dadour I.R. (2024). Morphological changes of larvae and pupae of *Lucilia sericata* (Diptera: Calliphoridae) reared at two temperatures and on three food types. J. Med. Entomol..

[17] Bauer A., Bauer A.M., Tomberlin J.K. (2020). Impact of diet moisture on the development of the forensically important blow fly *Cochliomyia macellaria* (Fabricius) (Diptera: Calliphoridae). Forensic Sci. Int..

[18] Stoffolano J.G., Gonzalez E.Y., Sanchez M., Kane J., Velázquez K., Oquendo A.L., Yin C.M. (2000). Relationship between size and mating success in the blow fly Phormia regina (Diptera: Calliphoridae). Ann. Entomol. Soc. Am..

[19] Saunders D.S., Bee A. (2013). Effects of larval crowding on size and fecundity of the blow fly, *Calliphorid vicina* (Diptera: Calliphoridae). EJE.

[20] Voss S.C., Cook D.F., Hung W.F., Dadour I.R. (2014). Survival and development of the forensically important blow fly, *Calliphora varifrons* (Diptera: Calliphoridae) at constant temperatures. Forensic Sci. Med. Pathol..

[21] Ong S.Q., Ahmad H., Tan E.H. (2018). Substrate moisture affects the development of *Megaselia scalaris* (Diptera: Phoridae): An implication of the growth circumstances of the fly in forensic entomology. Environ. Entomol..

[22] Kökdener M. (2021). Impact of diet and moisture content on the development of *Musca domestica* (Diptera: Muscidae). Environ. Entomol..

